# Low Molecular Weight Fucoidan Can Inhibit the Fibrosis of Diabetic Kidneys by Regulating the Kidney Lipid Metabolism

**DOI:** 10.1155/2021/7618166

**Published:** 2021-11-24

**Authors:** Yan Wang, Yanlei Sun, Fengli Shao, Bo Zhang, Zhen Wang, Xinpeng Li

**Affiliations:** ^1^College of Pharmacy, Linyi University, Linyi, Shandong, China; ^2^Linyi Tumor Hospital, Linyi, Shandong, China; ^3^College of Life Sciences, Linyi University, Linyi, Shandong, China; ^4^Chinese Academy of Traditional Chinese Medicine, China

## Abstract

In this study, a diabetic kidney disease model was established by placing the test rats on a high-sugar/high-fat diet combined with streptozotocin induction. Histopathological examination (H&E, Masson, and PASM stain) showed pathological changes in the diabetic rat kidneys, in addition to fibrotic symptoms and collagen deposition. Immunohistochemistry and western blot analyses indicated that the diabetic condition significantly increased the expressions of fibrotic markers including collagen, *α*-SMA, and fibronectin. The levels of cholesterol, triglyceride, and low-density lipoprotein were also increased in diabetic kidney disease (DKD) rat blood, while the level of high-density lipoprotein was decreased. The results of Oil red O staining experiments indicated that the kidneys of diabetic rats exhibited appreciable fat deposition, with high contents of triglyceride and cholesterol. To inhibit fibrosis and reduce fat deposition, low molecular weight fucoidan (LMWF) may be used. Based on PCR and western blot analyses, LMWF can regulate the expression levels of important lipid metabolism regulators, thereby impeding the development of kidney fibrosis. Through the vitro model, it also be indicated that LMWF could inhibit fibrosis process through regulating lipid metabolism which induced by palmitic acid.

## 1. Introduction

Diabetic kidney disease (DKD) is one of the leading causes of chronic kidney dysfunction. In China, DKD is more common than glomerular nephritis, and on a global level, DKD patients constitute one-third of all chronic kidney disease patients [1, 2]. According to the annual scientific report on kidney disease in China published in April 2019, DKD-induced uremia will peak in the next 10–20 years, which will heavily burden the medical and health systems in the country [3]. The incidence and mortality rates of chronic kidney disease are also expected to increase [1, 2], especially considering the scarcity of effective treatments and drugs.

In diabetes patients, continuous hyperglycemia may lead to microangiopathy, increased kidney vascular pressure, altered morphology and structure of the kidney, DKD, and ultimately, kidney fibrosis. Recent studies have shown that animals suffering from diabetes mellitus tend to develop DKD, and that once established, the disease causes lipid deposition in the kidneys. Depending on various factors, different degrees of lipid metabolism disorder can be observed in the kidneys of DKD animals [4, 5]. With time, the amount of accumulated lipids increases, and eventually, lipid toxicity damage is induced, which accelerates the development of kidney fibrosis [6]. Therefore, in order to prevent kidney fibrosis in DKD patients, the associated lipid metabolism disorder must be regulated [7]. In particular, the oxidation of triglyceride and the outflow of cholesterol should be controlled, as they are the main types of lipids that accumulate in the kidneys of DKD patients [8]. Based on previous studies, DKD fibrosis may also be delayed by regulating the mitochondrial uncoupling protein and enhancing the kidney tubular oxidation of fatty acids [9]. Overall, the available studies indicate that in order to prevent the progress of DKD into kidney fibrosis with time, the accumulated lipids should be effectively cleared [10, 11].

Low molecular weight fucoidan (LMWF) extracted from *Laminaria japonica* is a sulfated polysaccharide that is mainly composed of fucose. Fucoidan is known for its anti-inflammatory, antioxidation, and antifibrosis effects, as well as its capacity to protect kidneys against injury [12, 13]. In DKD rats, fucoidan effectively inhibits kidney damage and shows good therapeutic efficiency [14–16]. It also regulates the lipid metabolism disorder induced by diabetes [17, 18]. Lipid metabolism disorder is a common pathological feature of most diabetes. Triglyceride and cholesterol metabolism disorders are important inducements of DKD lipid metabolism disorder. Due to the imbalance of triglyceride oxidation and cholesterol outflow, they are deposited in the kidney of patients with DKD. Therefore, in order to regulate the ectopic deposition of renal lipids, it is necessary to effectively regulate the oxidation and transport of lipids in vivo. The decrease of fatty acid oxidation in patients with DKD destroys the balance between fatty acid synthesis, uptake, and consumption, resulting in intracellular lipid accumulation, so as to accelerate the development of DKD and aggravate the pathogenesis of renal fibrosis. Based on the regulation of fucoidan on lipid metabolism, the aim of this study was to determine whether LMWF can delay kidney fibrosis induced by diabetes through regulating lipid metabolism.

## 2. Materials and Methods

### 2.1. Reagents and Animals

Fucoidan was purchased from the Weihai Century Bokang Seaweed Co., Ltd., Shandong province, China. The extraction process is described as follows: The Laminaria japonica were sliced, crushed, and boiled in water (100°C). The aqueous solution was purified with the dialysis membrane (molecular weight cutoff, 3500 Da) against pure water. The dialysis fluid was concentrated and precipitated with ethanol (75% final concentration) [13, 19, 20].

Streptozotocin (STZ) was purchased from Sigma–Aldrich (St. Louis, USA; S0130-5G). All other chemicals and reagents were obtained from general commercial sources and used without prior treatment, unless otherwise specified. The antibodies were supplied by Affinity Bioscience Inc. (Cincinnati, OH, USA; Collagen: AF7001, Fibronectin: AF5335, *α*-SMA: AF1032), and the PCR kits were purchased from Thermo Fisher Scientific Inc. (Catalog: A25780). As for the triglyceride and cholesterol kits, they were obtained from Solarbio Life Science Co., Ltd. (Catalog: BC0625; BC1985). The experiments were performed on male SD rats (200 ± 20 g, SPFII Certificate) bought from Service Biotechnology Co., Ltd. (Wuhan, China). All experimental procedures were previously approved by the Animal Ethics Committee of Linyi University.

### 2.2. Preparation of Low Molecular Weight Fucoidan

The LMWF was prepared according to the method described in a previous study [14]. The chemical composition of LMWF (molecular weight = 8.84 kDa) is 28.03% fucose, 28.45% sulfate, and 8.31% uronic acid. The neutral sugar composition is 1 : 0.057 : 0.041 : 0.008 : 0.029 : 0.019 fucose: galactose: mannose: glucose: rhamnose: xylose (mole ratio).

### 2.3. Model Rat Groups

Male SD rats were randomly divided into three groups: Sham group (Sham), diabetic kidney disease (DKD) group, and low molecular weight fucoidan (LMWF) group; fosinopril (FP) was used as positive control group, with 10 rats in each group. All rats were subjected to light/dark cycles (12 : 12 h) for one week and fed with a high-sugar/high-fat diet (10% lard and 37% sucrose) for six weeks in order to produce insulin resistance, except the rats of the Sham group. On the seventh week, STZ was injected into DKD, FP, and LMWF rats for induction, whereas the Sham rats were administered with saline solution. Starting on the eighth week, the FP and LMWF groups were gavage fed with LMWF (200 mg/kg; dosage based on previous research [15, 16] and fosinopril (8 mg/kg/day) for 13 weeks). The Sham and DKD groups, in contrast, were given saline solution by gastric administration.

### 2.4. Assessment of Kidney Function

During the experiment, body weight changes and blood glucose levels were monitored regularly (once a week). Urine samples were collected on the 20th week using a metabolic cage. After measuring their volumes, the samples were analyzed for protein content. The urinary protein content was test with a kit (C035-2, Nanjing Jiangcheng Bioengineering Institute). After centrifugation, the supernatant of urine was put at 4°C for testing. The test process was carried out according to the instructions of the kit.

At the end of the experiment, blood samples were collected from all rats, and an automated chemistry analyzer was used to determine the blood urea nitrogen (BUN) and serum creatinine (Scr) levels in these samples. The body weights of rats were recorded before euthanasia; then, the kidneys were removed, washed with PBS, and weighed. The right kidney was immediately fixed in 4% formaldehyde for Hematoxylin and Eosin (H&E), Periodic Acid-Silver-Methenamine (PASM), and Masson staining in order to observe the histopathological alterations, morphological changes, and fibrosis in the glomerular mesangium and basement membrane. Immunohistochemistry and western blot analyses were used to detect the expressions of fibrosis biomarkers in the rat kidneys.

### 2.5. Evaluation of Lipid Metabolism in Kidneys

The triglyceride (TG), total cholesterol (TC), low-density lipoprotein (LDL), and high-density lipoprotein (HDL) levels in rat blood were determined at the end of the experiment. The kidney contents of triglyceride (TG) and total cholesterol (TC) were analyzed using the appropriate kits (Catalog: BC0625, BC1985; Solarbio, Beijing, China). First, the kidneys were ground with PBS and extracted with chloroform and methanol. Then, the sample was broken by ultrasound for 1 min, followed by incubation in a shaking incubator for 15 min. After centrifugation, the contents of triglyceride (TG) and total cholesterol (TC) were determined. Oil red O staining experiments were used to observe lipid deposition in the kidneys.

### 2.6. Detection of Lipid Metabolism Regulatory Factors in Kidneys

qPCR analysis was used to detect the mRNA expressions of regulatory factors related to triglyceride hydrolysis, triglyceride oxidation, and cholesterol efflux in kidney tissue. Total RNA was extracted from the kidney using Trizol (Invitrogen, Carlsbad, CA, USA; Catalog: 12183555). The purity was tested using Nanodrop, and the total RNA of each sample was reverse-transcribed into cDNA using the Revert Aid First Strand cDNA Synthesis Kit (Thermo; Catalog: K1621). Real-time PCR was performed on an ABI-Prism 7500 using Power SYBR Green (Thermo Fisher Scientific Inc., Catalog: A25780). The comparative Ct method was used to quantify gene expression, and the relative quantification was calculated as 2^−△△Ct^. The presence of nonspecific amplification products was excluded by melting curve analysis [13].

The primers are as follows: Peroxisome proliferators-activated receptor-*α* (PPAR-*α*): F 5′-GGCTCGGAGGGCTCTGTCATC-3′, R 5′-ACATGCACTGGCAGCAGTGGA-3′; Carnitine acyltransferase-1 (CPT-1): F 5′-TTCACTGTGACCCCAGACGGG-3′, R 5′-AATGGACCAGCCCCATGGAGA-3′; Liver X receptor (LXR-*α*): F 5′-TCTGGAGACATCTCGGAGGTA-3′, R 5′-GGCTCACCAGTTTCATTAGCA-3′; ATP-binding cassette transporter A1 (ABCA1): F 5′-GAACTGGCTGTGTTCCATGAT-3′, R 5′-GATGAGCCAGACTTCTGTTGC-3′; Apolipoprotein E (ApoE): F 5′-ATGAAGGTTCTGTGGGTTGC-3′, R 5′-GCTGCTGGCGTAACTTATCC-3′; Peroxisome proliferator-activated receptor *γ* coactivator-1 (PGC-1): F 5′-TCAGTCCTCACTGGTGGACA-3′, R 5′-TGCTTCGTCAAAAACAG-3′; CD36: F 5′-GGTGTGCTCAACAGCCTTATC-3′, R 5′-TTATGGCAACCTTGCTTATG-3′; and *β*-actin: F 5′-TTGTAACCAACTGGGACGATATG-3′, R 5′-GATCTTGATCTCATGGTGCTAGG-3′.

The protein expressions were determined using western blot analyses. The liver was washed with ice-cold PBS, then weighed (~100 mg) and cut into small pieces, and suspended in 200 *μ*L NP-40 lysis buffer (Beyotime Biotechnology, Shanghai, China; P0013F). The liquid supernatant was collected after centrifugation at 12,000 × g at 4°C for 10 min. Then, the total protein concentration was determined using a BCA protein assay kit (Beyotime Biotechnology, Shanghai, China; P0010S). After adding 5x loading buffer (Solarbio Life Science Co., Ltd.; P1040), the proteins were boiled at 99°C for 10 min, after which they were resolved on SDS-PAGE gels, electro transferred to PVDF membranes, blocked in 5% milk in TBST at room temperature for 1 h, and then incubated with primary antibodies at 4°C overnight. Then, the membranes were incubated with horseradish peroxidase-conjugated secondary antibody at room temperature for 1 h. Chemiluminescent reagents were used to visualize the protein blots, and *β*-actin served as the loading control [13].

### 2.7. Effect of LMWF on HK-2 Cells and HBZY-1 Cells

The HK-2 cell line (human renal proximal tubular cell line) and NBZY-1 cells (Rat glomerular mesangial cells) were grown in DMEM/F-12 (17.5 mM glucose; Hyclone, Logan, UT, USA) supplemented with 10% fetal bovine serum (Gibco, Grand Island, NY, USA), 2 mM L-glutamine, 100 U/mL penicillin, and 100 *μ*g/mL streptomycin. Cells were cultured to subconfluence at 37°C in a 5% CO2 water-saturated atmosphere. When 90% confluency was reached, the cells were transferred to 96-well plates, with 2 × 10^3^ cells in each well. Following 24 h starvation in a serum-free medium, the cells were treated with LMWF (10-1000 *μ*g mL^−1^) and cultured for 72 h. Other cells were cultured for 72 h with 30 mmol L^−1^ glucose and 10, 20, or 40 *μ*g mL^−1^ LMWF. The viability of the cultured cells was measured using the CCK-8 kit, and the optimum LMWF concentration was determined.

The cells were cultured for 72 h in six-well plates, and they were divided into four groups: normal glucose (NG), high glucose (HG), high glucose + palmitic acid (HG + PA), and high glucose + palmitic acid+ LMWF (HG + PA + LMWF) groups. Western blot analyses were conducted in order to express/quantify proteins in HK-2 cells and HBZY-1 cells, as detailed in a previous study [13]. Then, it was analyzed by chemiluminescence, using *β*-actin as a loading control, to visualize the proteins.

### 2.8. Statistical Analysis

All quantitative data presented in this text are expressed as mean ± SD. Statistical analysis was carried out using the GraphPad Prism 8.0 software, and variations among groups were assessed using one-way analysis of variance (ANOVA) followed by Dunnett's test. Differences were considered to be statistically significant and very significant at *p* < 0.05 (∗) and *p* < 0.01 (∗∗), respectively.

## 3. Results

### 3.1. Effect of Low Molecular Weight Fucoidan on Kidney Fibrosis

Some DKD and LMWF rats died late in the feeding period. The mortality rates in the DKD and LMWF groups were found to be 30%, 20%, and 20% in the FP group. Blood glucose analyses show that the glucose levels in the three groups were higher than 16.5 mmol L^−1^, the prescribed value of the diabetic model. The Sham group rats had smooth and bright hair, compared to dry and lusterless hair for DKD rats. As for the FP and LMWF rats, their hair was like that of Sham group rats.

**Figure 1:**
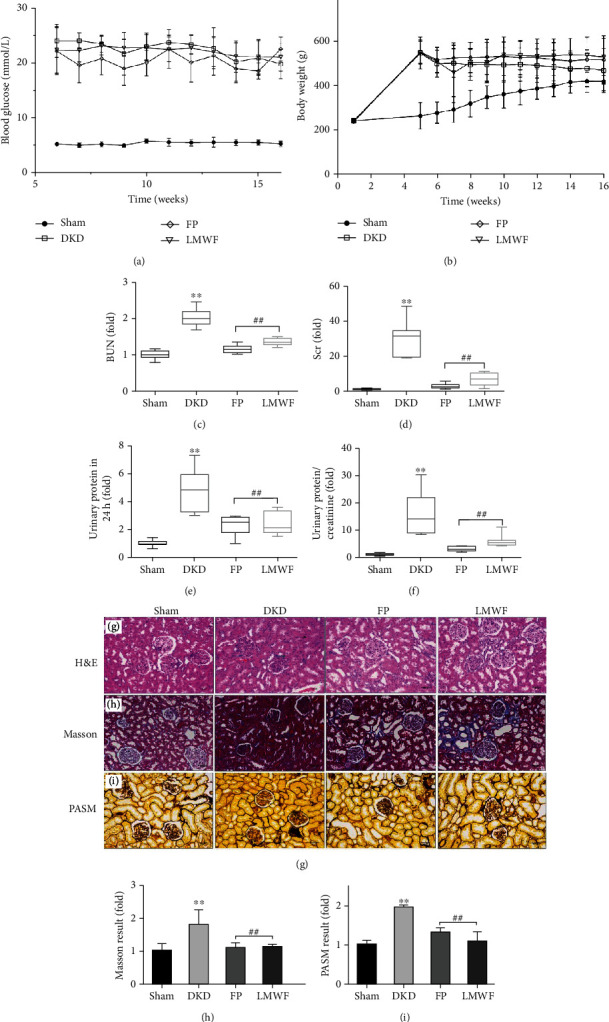
(a) Variations in body weight levels. (b) Variations in blood glucose levels. Variations in (c) BUN, (d) Scr, (e) urinary protein (24-hour urine), and (f) protein/Scr levels at the end of the experiment (^∗∗^*p* < 0.01 vs. the Sham group; ##*p* < 0.01 vs. the DKD group, *n* ≥ 6). (g) Hematoxylin and Eosin staining to observe kidney injury in rats (black arrow: dilation of kidney tubules; green arrow: mesangial matrix hyperplasia; red arrow: eosinophils; purple arrow: glomerular hypertrophy). (h) Masson staining to observe kidney fibrosis in rats. (i) Periodic Acid-Silver-Methenamine staining to observe collagen deposition in rats. (j) Masson staining. (k) Statistical analysis results. The statistical results were compared with the Sham operation group and other groups.

From Figure 1(a), we found that the body weight of rats in the Sham group increased continuously, while the rats in the DKD group showed a downward trend after modeling. LMWF and fosinopril can control the body weight of rats in a relatively stable state. After modeling, the blood glucose inhibition of DKD group was maintained at a high level, and LMWF and fosinopril did not show the effect of reducing blood glucose (Figure 1(b)). Figures 1(c) and 1(d) show the levels of blood urea nitrogen (BUN) and serum creatinine (Scr), two important indicators of kidney function, measured at the end of the experiment. Compared to the Sham group, the rats in the DKD group present significantly increased BUN and Scr levels (the fluctuation of serum creatinine was relatively large, but it showed the differential expression). In the FP and LMWF groups, these levels were appreciably lower than those detected in the DKD group, which indicates that LMWF can effectively regulate the kidney damage caused by diabetes, and the effect was similar to fosinopril. This is consistent with the pathological observation results discussed previously. The concentration of urine protein was determined in 24-hour urine samples collected in a metabolic cage. The measured values indicate that DKD rats exhibit the highest content of urine protein (Figure 1(e)). Moreover, the ratio of urinary protein to creatinine in these rats is significantly higher than that detected in Sham and LMWF group rats (Figure 1(f)). This suggests that the kidney damage observed in the DKD group may be caused by diabetes.

The Hematoxylin and Eosin (H&E) staining results presented in Figure 1(g) demonstrate that DKD rats exhibit mesangial matrix hyperplasia, glomerular hypertrophy, dilation of a small number of kidney tubules, and epithelial cell edema. Collagen deposition and fibrosis symptoms are also evident in the kidney cells of these rats, as shown in the images recorded after Periodic Acid-Silver-Methenamine (PASM) and Masson staining, respectively (Figures 1(i) and 1(h)). Masson and PASM staining were analyzed by the Image-Pro 6.0 software. The results showed that the collagen distribution in the DKD group was significantly higher than that in the Sham operation group (Figures 1(j) and 1(k)). Comparatively, the LMWF group exhibits less severe effects, which indicates that fucoidan can relieve the kidney damage caused by DKD. At the same time, we could also observe that fosinopril can effectively inhibit the process of renal fibrosis in diabetic rats.

### 3.2. Expression of Fibrotic Biomarkers

**Figure 2:**
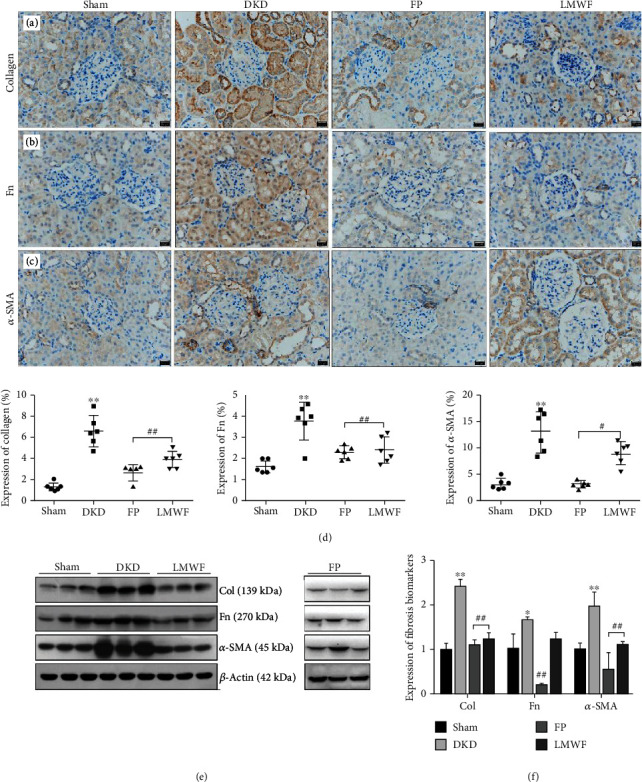
Assessment of kidney fibrosis. Immunohistochemical detection of fibrosis biomarkers: (a) collagen-1, (b) fibronectin (Fn), and (c) *α*-SMA. (d) Statistical expressions of fibrosis biomarkers in the immunohistochemical assay (^∗∗^*p* < 0.01 vs. the Sham group; ##*p* < 0.01 vs. the DKD group, *n* = 6). (e) Detection of fibrosis biomarker protein expressions by western blot analysis. (f) Statistical expressions of fibrosis biomarkers in the western blot assay (^∗^*p* < 0.05, ^∗∗^*p* < 0.01 vs. the Sham group; ##*p* < 0.01 vs. the DKD group, *n* = 3). The statistical results were compared with the Sham operation group and other groups.

The results of immunohistochemistry analyses demonstrate that the expression levels of fibrosis biomarkers, including collagen I, fibronectin, and *α*-SMA protein, are significantly higher in the kidneys of DKD rats than in Sham group rats (Figures 2(a)–2(c)). The western blots presented in Figures 2(e) and 2(f) confirm that collagen 1, *α*-SMA protein, and fibronectin (Fn) are highly expressed in the diabetic rat model, and that the expressions of these biomarkers decrease significantly upon the administration of LMWF.

### 3.3. Effect of LMWF on Diabetes-Induced Kidney Lipid Metabolism

**Figure 3:**
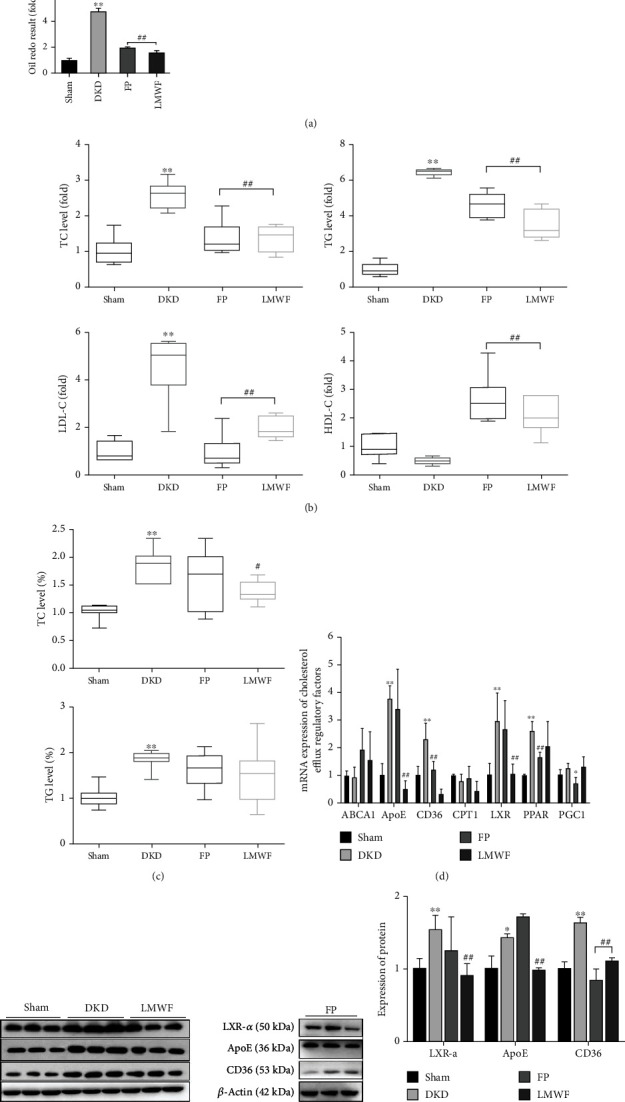
Assessment of lipid metabolism in rat kidneys. (a) Oil red O staining to observe lipid deposition in the kidneys of rats and Oil red O staining statistical analysis results. (b) Serum biochemical analysis: variations in TC, TG, LDL-C, and HDL-C at the end of the experiment (^∗∗^*p* < 0.01 vs. the Sham group; ##*p* < 0.01 vs. the DKD group, *n* ≥ 6). Detection of (c) TC and (d) TG contents in the kidneys of rats (^∗∗^*p* < 0.01 vs. the Sham group; ##*p* < 0.01 vs. the DKD group, *n* ≥ 6). (d) Variation of regulatory factor mRNA expressions. (e) Detection of regulatory factor protein expressions by western blot. (f) Statistical protein expressions of the regulatory factors of lipid metabolism (^∗^*p* < 0.05, ^∗∗^*p* < 0.01 vs. the Sham group; ##*p* < 0.01 vs. the DKD group, *n* = 3). The statistical results were compared with the Sham operation group and other groups.

The Oil red O staining results illustrated in Figure 3(a) show that the kidneys of DKD rats exhibit substantial fat deposition, compared to almost no fat deposition in the Sham group (fat deposition was analyzed by the Image-Pro 6.0 software). The administration of LMWF significantly reduces the elevated fat deposition levels detected in DKD rat kidneys. Based on the blood biochemical analysis results, the diabetic model (DKD rats) shows increased levels of total cholesterol (TC), triglyceride (TG), and low-density lipoprotein (LDL), and a decreased level of high-density lipoprotein (HDL) relative to the Sham group rats. LMWF treatment reverses these undesirable effects of diabetes (Figure 3(b)). The triglyceride and cholesterol contents in the extracted kidney fat of the DKD group were found to be much higher that of the Sham group. As for the LMWF and FP groups, it shows effectively reduced TC content compared to DKD; however, LMWF treatment seems to have no appreciable effect on TG deposition. Therefore, it may be concluded that LMWF cannot inhibit the deposition of cholesterol in the kidneys of diabetic rats (Figure 3(c)).

### 3.4. Expressions of Kidney Lipid Metabolism Regulators

At the end of the experiment, the RNA expressions of kidney lipid metabolism regulators were determined using qPCR analysis. Compared to Sham group rats, the expressions of ApoE, CD36, and LXR in DKD were found to be changed. The administration of LMWF helps to restore the RNA expressions of these lipid-metabolism-related regulators to their normal values (Figure 3(d)). The protein expressions of the regulators were determined using protein immunoblotting experiments. The western blots presented in Figures 3(e) and 3(f) show that diabetes significantly increases the expressions of ApoE, CD36, and LXR in the kidneys of DKD rats, while LMWF treatment decreases them. This indicates that LMWF can effectively reduce the deposition of fat in the kidneys of DKD rats by regulating the expressions of the regulatory factors related to lipid deposition. At the same time, we also found that fosinopril had no effect on ApoE and CD36.

### 3.5. Effect of LMWF on HK-2 Cells and HBZY-1 Cells

**Figure 4:**
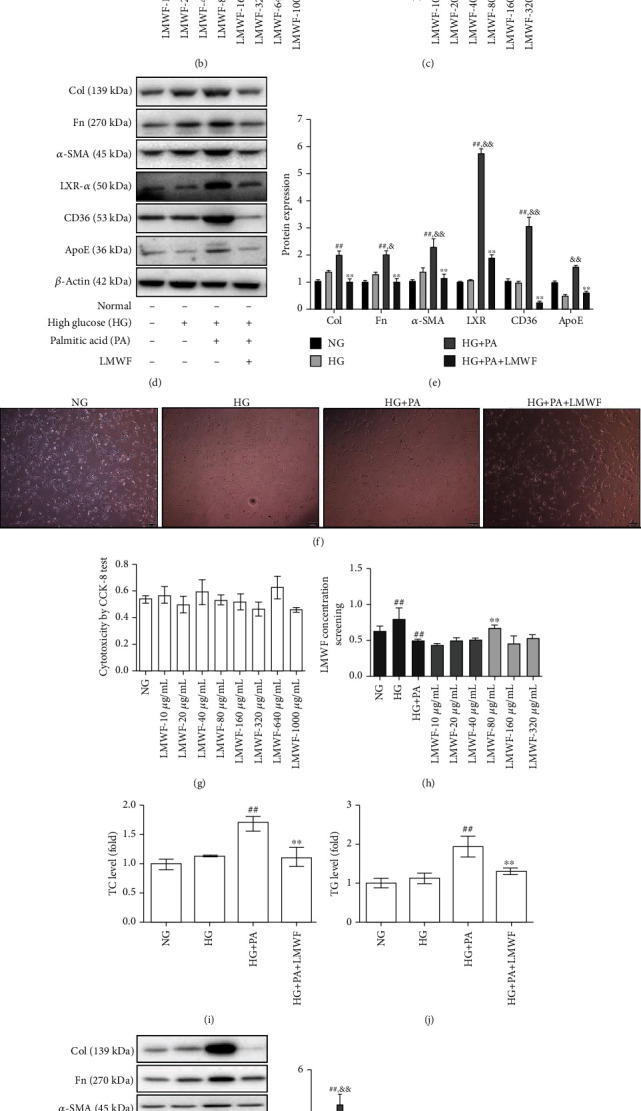
Assessment of lipid metabolism in HK-2 cells. (a) Observing the state of HK-2 cells. (b) Cytotoxicity of LMWF effect on HK-2 cells. (c) LMWF concentration screening (^∗∗^*p* < 0.01 vs. the HG + PA group; ##*p* < 0.01 vs. the NG group, repeat three times). (d) Detection of fibrosis biomarkers and regulatory factor protein expressions by western blot. (e) Statistical protein expressions of fibrosis biomarkers and the regulatory factors of lipid metabolism (^∗∗^*p* < 0.01 vs. the HG + PA group; ##*p* < 0.01 vs. the NG group; &*p* < 0.05, &&*p* < 0.01 vs. the HG group, repeat three times). (f) Observing the state of HBZY-1 cells. (g) Cytotoxicity of LMWF effect on HBZY-1 cells. (h) LMWF concentration screening (^∗∗^*p* < 0.01 vs. the HG + PA group; ##*p* < 0.01 vs. the NG group, repeat three times). (i) TC and (j) TG contents in the HBZY-1 cells (^##^*p* < 0.01 vs. the Sham group; ^∗∗^*p* < 0.01 vs. the DKD group, *n* ≥ 6). (k) Detection of fibrosis biomarkers and regulatory factor protein expressions in HBZY-1 cells by western blot. (l) Statistical protein expressions of fibrosis biomarkers and the regulatory factors of lipid metabolism (^∗∗^*p* < 0.01 vs. the HG + PA group; #*p* < 0.05, ##*p* < 0.01 vs. the NG group; &*p* < 0.05, &&*p* < 0.01 vs. the HG group, repeat three times). The statistical results were compared with the Sham operation group and other groups.

An in vitro diabetic lipid metabolism model was established by stimulating HK-2 cells and HBZY-1 cells with high glucose and palmitic acid (Figures 4(a) and 4(f)). In the experiment, LMWF did not show toxic effect on HK-2 cells and NBZY-1 cells (Figures 4(b) and 4(g)). In the process of cell culture, the cell state of HG + PA group was significantly different from that of NG group, and the number of cells was significantly reduced. After that, the concentration of LMWF was screened. The experimental results showed that the effect of 40 *μ*g/mL (HK-2 cells) and 80 *μ*g/mL (HK-2 cells) LMWF was the closest to the NG group. However, there was no significant difference between high concentration LMWF group and HG + PA group. When the concentration of LMWF was lower than 40 *μ*g/mL and 80 *μ*g/mL, the difference was found in the HG group, so the concentration of 40 *μ*g/mL and 80 *μ*g/mL were taken as the experimental concentration (Figures 4(c) and 4(h)).

After 72 hours of culture, cell proteins were collected; the TC, TG kits, and western blot were used to detect the content of TC, TG in HBZY-1 cells, and expression of fibrosis markers and lipid regulatory factors. In the results, we found that high glucose combined with palmitic acid stimulation can significantly enhance the content of TC and TG in HBZY-1 cells (Figures 4(i) and 4(j)). At the same time, the expression of fibrotic markers (collagen, Fn, *α*-SMA) increased, which indicates that lipid metabolism deposition can accelerate the progress of fibrosis. The expression of lipid metabolism regulatory factor CD36 was also enhanced. LMWF could effectively reduce the expression of fibrosis markers and lipid metabolism regulatory factors, but the HG group compared with the NG group, fibrosis markers did not show difference (Figures 4(k) and (l)).

## 4. Discussion

Recently, research in the field of diabetes mellitus has revealed that kidney fibrosis symptoms caused by DKD are closely related to lipid metabolism. Diabetic rats kept in a state of hyperglycemia for a long time show disorders in the functions of fat synthesis and liver transport, which eventually leads to the ectopic deposition of fat in the kidneys. According to numerous available studies, fat deposition in the kidneys is an important factor affecting kidney fibrosis [21, 22]. Previously, it had been shown that LMWF can effectively inhibit DKD-induced kidney fibrosis. However, is this process related to the regulation of lipid metabolism? Can LMWF delay renal fibrosis by regulating lipid metabolism?

In the study, we investigate the relationship between DKD-induced fibrosis and lipid metabolism disorder by analyzing the effects of LMWF on the kidneys and fat levels in type 2 diabetic rats. The obtained results demonstrate that DKD rats exhibit obvious symptoms of kidney fibrosis, and that the expressions of fibrosis markers in these rats are significantly increased. Moreover, blood biochemical analysis and red O staining confirm that lipids accumulate in the kidneys of the diabetic rats. The Sham group rats, in contrast, show no obvious lipid deposition or fibrosis. Therefore, it may be concluded that DKD-induced kidney fibrosis is indeed related to kidney lipid deposition.

Like previous studies, our results also show that LMWF can inhibit kidney fibrosis [13, 20]. Histopathological examinations demonstrate that fucoidan effectively alleviates the symptoms of fibrosis. Moreover, it significantly reduces the expression levels of fibrosis biomarkers, as evidenced by immunohistochemistry and western blot analyses. Moreover, compared with the fosinopril treatment group, the treatment effect of LMWF was also very well; there was no significant difference between the two. LMWF can also decrease the blood levels of TC, TG, and LDL in DKD rats while increasing the level of HDL. Based on the recorded images of the Oil red O staining experiment, fat deposition in the kidneys of diabetic rats decreases upon treatment with LMWF. The increased kidney content of TC in DKD rats may also be reduced by LMWF; however, fucoidan has practically no effect on the content of TG. Overall, our results suggest that LMWF can decrease the deposition of lipids in the kidneys of DKD rats by regulating TC efflux. To confirm this conclusion, the mRNA and protein expressions of regulatory factors related to TC efflux were determined [23]. Interestingly, we found that the expressions of ABCA1 and CPT-1 in DKD rat kidneys are the same as those in normal rats [24]. However, significant changes were detected in the expressions of PPAR-*α* and PGC-1 [25]. LMWF treatment does not affect any of the aforementioned factors, but it obviously affects the expressions of LXR-*α*, ApoE, and CD36.

In vitro model experiments, we found that the fibrosis markers of HK-2 cells and HBZY-1 cells under high glucose condition were not obvious, but the expression of fibrosis markers was significantly increased after combined with palm stimulation, and some fibrosis markers were also appeared. This also shows that lipid metabolism disorder will accelerate the development of fibrosis; LMWF could delay the fibrosis process which caused by lipid metabolism disorder. However, the fibrosis biomarkers after high glucose stimulation only were not particularly obvious. It may be that the high glucose stimulation needs a longer duration of time, and the time of cell culture in vitro was not enough. In addition, we also found that LMWF could effectively reduce the expression of lipid metabolism regulatory factors, which was consistent with previous experimental results in vivo, which also proved that LMWF can delay the process of renal fibrosis by regulating the expression of lipid metabolism-related factors.

As an oxidative sterol activated nuclear receptor, LXR-*α* regulates the expression of some key genes in the process of TC metabolism [26]. It is also involved in the regulation of other physiological activities, including fat formation, glucose metabolism, macrophage innate immunity, and inflammatory response [27]. ApoE is a target gene of LXR-*α* in adipose tissue, and so, it is directly regulated by LXR-*α* [28]. Clinically, high ApoE expressions are indicative of dyslipidemia, a condition characterized by elevated levels of TC and TG in the plasma [29]. The increased ApoE expression detected in the kidneys of rats investigated in this study is thus evidence that these rats suffer from lipid metabolism disorder. The long-term hyperglycemia condition in the rats eventually leads to liver injury and to the accumulation of fat in the kidneys [30]. CD36 is also a target gene of LXR-*α* and can be directly regulated by it [31]. The available studies confirm that CD36 plays an important role in the process of kidney lipid deposition by regulating the uptake of cholesterol [32–34]. Many ligands on kidney cells can bind with CD36 to form ligand complexes that can activate regulatory factors such as protein kinase C- (PKC-) NAPDH oxidase and TGF-*β*1 [35, 36]. The complexes affect PKC activity, inflammatory response, oxidative stress reaction, and TGF-*β*1 secretion, and so, they have a significant influence on the development of fibrosis. As shown herein, LMWF treatment significantly inhibits the expression of CD36, thereby restricting the formation of ligand compounds and blocking the processes of inflammation and fibrosis. Based on previous studies, LMWF can also alleviate renal fibrosis by regulating the CD36 factor [13, 37]. Considering that LMWF can simultaneously regulate the expressions of LXR-*α*, ApoE, and CD36, and that ApoE and CD36 are target genes of LXR-*α*, it may be speculated that LMWF actually regulates the expression of LXR-*α* only, which in turn affects the expressions of ApoE and CD36.

## Data Availability

The data used to support the findings of this study are included within the article.

## Abbreviations

DKD: Diabetic kidney disease
LMWF: Low molecular weight fucoidan
STZ: Streptozotocin
FP: Fosinopril
H&E: Hematoxylin-eosin staining
PASM: Periodic Acid-Silver-Methenamine staining
BUN: Blood urea nitrogen
Scr: Serum creatinine
TG: Total triglyceride
TC: Total cholesterol
LDL: Low-density lipoprotein
HDL: High-density lipoprotein
HG: High glucose
PA: Palmitic acid.
